# The True Physician

**Published:** 1880-09

**Authors:** 


Is sadly forgetful of his high mission, who supposes that his aim and duty end in relieving the ailments of those who seek his assistance from day to day; that should be regarded as only the means to higher ends, the means of experience and wisdom and leisure to institute measures for the discovery and removal of causes, which, in their silent and stealthy progress, have in all past time, eaten out the vitality of families, and nations, and races, which once lived in glory, but of which, the habitable globe affords not now a single living relic.

